# Self-reported quality of recovery after radical prostatectomy—a prospective cohort study

**DOI:** 10.1007/s11136-025-04026-6

**Published:** 2025-07-18

**Authors:** Marlene Fischer, Josephine Küllmei, Linda Krause, Peipei Wei, Ursula Kahl, Elena Kainz, Caspar Mewes, Markus Graefen, Alexander Haese, Christian Zöllner, Lili Plümer

**Affiliations:** 1 Department of Anesthesiology, University Medical Center Hamburg-Eppendorf, Hamburg, Germany; 2 Department of Intensive Care Medicine, University Medical Center Hamburg-Eppendorf, Martinistrasse 52, 20246 Hamburg, Germany; 3 Institute of Medical Biometry and Epidemiology, University Medical Center Hamburg-Eppendorf, Hamburg, Germany; 4 Martini-Klinik, Prostate Cancer Center, University Medical Center Hamburg-Eppendorf, Hamburg, Germany

**Keywords:** Quality of recovery, Quality of care, Standardized endpoint, Perioperative medicine, Patient-reported outcome, Non-cardiac surgery

## Abstract

**Purpose:**

The Quality of Recovery-15 questionnaire (QoR-15) has been developed to assess patient-reported recovery 24 h after non-cardiac surgery. This prospective cohort study sought to analyze patient-reported recovery throughout day five after open radical retropubic prostatectomy (ORP) and robot-assisted radical retropubic prostatectomy (RARP).

**Methods:**

Between June 2022 and February 2023 adult patients, who were scheduled for elective radical prostatectomy, completed the German version of the QoR-15 (QoR-15GE) preoperatively to establish a baseline value. Between postoperative day one and day five, patients completed the QoR-15GE daily until the day of discharge.

**Results:**

A total of 523 patients completed the questionnaires. On postoperative day one QoR-15GE scores were significantly lower after RARP compared with ORP (ORP: 113 ± 22 vs. RARP: 107 ± 24; *p* = 0.006) with a higher decline in postoperative QoR-15GE scores in RARP compared with ORP patients (ORP: 27 ± 20 vs. RARP: 32 ± 23; *p* = 0.006). The multivariable analysis confirmed an influence of surgical technique (Estimate: 4.39; 95% CI [0.27; 8.50], *p* = 0.037) on postoperative quality of recovery after adjusting for clinically relevant variables. Irrespective of surgical technique, we observed a consistent increase in QoR-15GE scores with similar recovery scores on postoperative days three, four, and five.

**Conclusion:**

Patients who undergo RARP experience poorer postoperative recovery at postoperative days one and two compared to those undergoing ORP. However, recovery scores align from postoperative day three, indicating a similar level of patient-reported recovery before hospital discharge. These findings suggest that the QoR-15GE may be appropriate for serial assessments.

**Supplementary Information:**

The online version contains supplementary material available at 10.1007/s11136-025-04026-6.

## Background

Ensuring patient satisfaction and promoting well-being before, during, and after surgical procedures stands as a primary goal of all medical specialties involved in perioperative care [1]. However, the quality of recovery after surgery is often solely assessed through objective parameters like morbidity, mortality, and the occurrence of complications during the perioperative period [2]. By contrast, subjective aspects such as pain, nausea or vomiting, sleep quality, the ability to mobilize or eat, and general physical and psychological well-being after surgery remain underreported [2].

Prostate cancer is the most common malignant disease in men in Germany and it is the second most commonly occurring cancer worldwide [3, 4]. Radical prostatectomy is one treatment option for patients with intermediate- or high-risk disease [5]. Approaches for the surgical treatment of prostate cancer include: 1) robot-assisted (RARP) and 2) open retropubic (ORP) radical prostatectomy, which do not differ in terms of oncologic or functional urological outcomes [6–9]. There is mounting evidence that the rate of postoperative complications is lower after RARP compared with ORP [10, 11]. However, there is an ongoing debate, on whether RARP beneficially affects patient-reported postoperative outcomes [12, 13]. While functional urological outcomes and quality of life have been studied extensively after surgical treatment for prostate cancer, overall self-reported health status in the immediate postoperative period remains underreported [6, 14]. In this context, it is important to investigate the impact of the surgical technique on self-reported recovery before hospital discharge.

Quality of Recovery (QoR) scales were developed to systematically assess the postoperative well-being and recovery perceived by patients [15, 16]. The application of QoR scales has been recommended for the evaluation of patient-reported outcomes in the postoperative phase [1]. In this study, we aimed to assess the impact of the surgical procedure – RARP vs. ORP – on self-reported recovery from the first day after surgery until hospital discharge. The secondary aim of this study was to analyze repeated assessments of the QoR-15GE throughout day five post-surgery.

## Methods

### Ethical information

Ethical approval for this study (reference number: 2022-100812-BO-ff) was obtained from the Ethics Committee at the Hamburg Chamber of Physicians on May 03, 2022. All study participants provided their written informed consent before the initiation of study-related procedures. The study and all related procedures have been performed in accordance with the World Medical Association’s Declaration of Helsinki.

### Design, setting and participants

We performed a prospective cohort study at the Department of Anesthesiology of a tertiary care university hospital and the Martini Clinic, a large prostate cancer center, in Northern Germany. Between June 2022 and February 2023, we screened patients scheduled for elective radical prostatectomy. Patients were eligible for inclusion if they were 18 years of age or older and had sufficient proficiency in German language to complete the questionnaire. Exclusion criteria were cognitive impairment requiring a legal guardian, ambulatory surgery, or planned postoperative transfer to the intensive care unit.

### Data collection

In 2013, the QoR-15 questionnaire was developed to assess the quality of recovery after surgery [16]. Containing 15 items, the QoR-15 addresses five dimensions of postoperative health status: psychological support, physical comfort, emotional state, physical independence, and pain. Each item may be rated on an 11-point Likert scale ranging from 0 to 10. Results from single items are summed up to one total score that can reach a maximum of 150 points. Higher scores indicate better recovery. Indicating a relevant change in postoperative health status, the minimal clinically important difference has been determined at 6 points [17]. We had previously translated and psychometrically evaluated a German version of the QoR-15 questionnaire (QoR-15GE), which was used for this study [18]. To establish baseline values, all study participants were asked to complete the QoR-15GE questionnaire one day before surgery, following the recommendation by Stark et al. [16]. Postoperatively, patients completed the QoR-15GE between day one and day five after surgery or until discharge, whichever occurred first. All patients received oral and written instructions upon first completion of the questionnaire and filled in paper versions of the QoR-15GE.

During the preanesthetic consultation, patients were interviewed regarding their demographic characteristics, medical history, and medication history. Information concerning variables pertaining to anesthesia and surgery was retrieved from the electronic anesthesia protocols and patient records.

### Hypotheses and endpoint

The primary endpoint was the difference in QoR-15GE scores between baseline assessments and the assessments one day after surgery. We hypothesized that patients undergoing RARP would have better recovery, reflected by a lower decline in QoR-15GE scores (∆QoR-15GE) between assessments at baseline and on the first postoperative day, compared with patients undergoing ORP. Our second hypothesis was that the QoR-15GE mirrors postoperative quality of recovery beyond the first postoperative day and is suitable for serial assessments.

### Sample size

Based on the minimal clinically important difference, which has been determined at 6 points for the QoR-15, a difference of 6 points or higher between surgical procedures was considered clinically relevant [17]. At the time of study conception, ORP and RARP were performed at a ratio of 1:2 at our center. Therefore, 174 (ORP) and 348 (RARP) patients are required in order to detect a difference of 6 points between the two surgical procedures with a power of 80%, a significance level of 5% and the use of a two-sided two-sample t-test.

### Statistical analysis

Data are reported as mean ± standard deviation or as numbers and percentages, as appropriate. The standardized response mean was computed by dividing the difference between the means of pre- and postoperative QoR-15GE scores by the standard deviation of the differences. The total change in QoR-15GE scores between the preoperative baseline values and the first postoperative day (ΔQoR-15GE) were compared between patients undergoing ORP and RARP using the two-sided two-sample t-test.

Multivariable linear regression analysis was performed to assess the influence of the surgical procedure on ΔQoR-15GE while taking into account potentially confounding variables. Aside from the independent variable of primary interest, ‘surgical technique’, the following variables were included in the model: ‘preoperative QoR-15GE score’, ‘duration of surgery (min)’, ‘nerve resection (yes/no)’, ‘Charlson Comorbidity Index’. All statistical analyses were conducted using R Statistical Software (version 3.5.3; R Foundation for Statistical Computing, Vienna, Austria).

To assess whether self-reported quality of recovery shows differing temporal developments by surgical technique, we calculated a linear mixed effects model including an interaction term between the time points of assessment and surgical technique. We used a linear mixed effects model with random intercepts for the patients to account for the repeated measurement structure of the data and the Kenward-Roger method to estimate the denominator degrees-of-freedom. We report the model results using estimated marginal means, contrasts and corresponding 95% confidence intervals. As an additional analysis, the same model was recalculated including the potentially confounding variables described above. Analyses were performed for complete cases only, since missing data were not imputed.

## Results

**Fig. 1:**
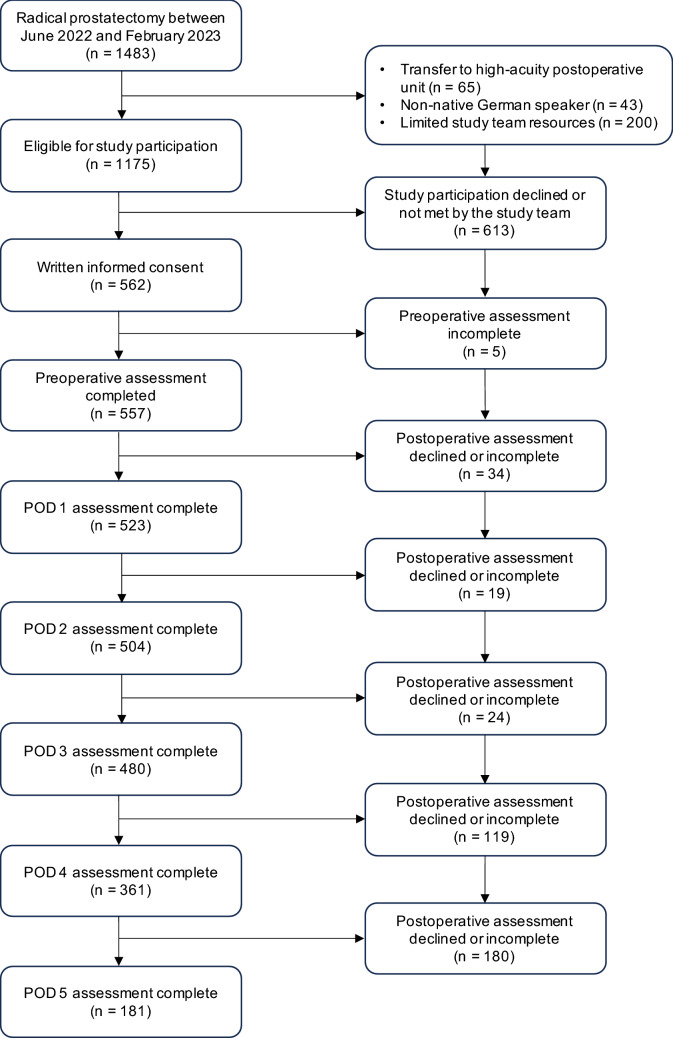
The flow diagram shows the flow of participants throughout the study. *POD* postoperative day

Between June 2022 and February 2023, at total of 562 patients were included. Of these, 523 completed the questionnaires preoperatively and one day after surgery, resulting in a completion rate of 93%. Figure 1 shows the flow of participants throughout the study. Patient characteristics, surgical variables, and anesthesia-related information are presented in Table 1.

**Table 1:** Demographic and clinical characteristics of the study population

	ORP (n = 162)	RARP (n = 400)	*P*	Missing (n)
Age, years	65 ± 6	63 ± 7	0.002	
Body Mass Index, kg*m^−2^	26.4 ± 2.9	27.1 ± 3.8	0.037	
Arterial hypertension	78 (48.1)	184 (46.0)	0.712	
Obstructive sleep apnea	42 (25.9)	74 (18.5)	0.064	
CCI total score	5 ± 2	5 ± 1	0.001	
ASA physical status			0.083	
I	11 (6.8)	24 (6.0)		
II	114 (70.4)	315 (78.8)		
III	37 (22.8)	61 (15.2)		
Sevoflurane	71 (43.8)	389 (97.2)	0.001	
Propofol	99 (61.1)	48 (12.0)	0.001	
Sufentanil, µg	89 ± 17	93 ± 47	0.206	
Duration of anesthesia, min	250 ± 50	248 ± 37	0.601	
Crystalloid fluids, ml	4062 ± 807	4015 ± 641	0.469	
Noradrenaline^a^, µg*kg^−1^*min^−1^	0.11 ± 0.10	0.12 ± 0.06	0.012	
Duration of surgery, min	175 ± 44	174 ± 41	0.902	
Blood loss, ml	747 ± 430	293 ± 214	0.001	
Pain intensity^b^	3.4 ± 2.0	3.5 ± 2.0	0.859	
Piritramide, mg	5.0 ± 4.8	3.8 ± 3.6	0.001	
Ondansetron	3.2 ± 1.7	3.5 ± 1.6	0.135	
PACU length of stay, min	184 ± 72	171 ± 54	0.014	
Neoadjuvant treatment	4 (2.5)	8 (2.0)	0.979	
pT stage^c^			0.046	1
2a	3 (1.9)	23 (25.8)		
2c	88 (54.3)	233 (58.4)		
3a	36 (22.2)	91 (22.8)		
3b	34 (21.0)	51 (12.8)		
No histological tumor detection	1 (0.6)	1 (0.3)		
N stage^c^			0.082	1
0	122 (75.3)	328 (82.2)		
1	40 (24.7)	71 (17.8)		
UICC			0.017	1
0	1 (0.6)	1 (0.3)		
1	3 (1.9)	18 (4.5)		
2	86 (53.1)	232 (58.1)		
3	41 (25.3)	110 (27.6)		
4	31 (19.1)	38 (9.5)		
Nerve resection			< 0.001	1
None	111 (68.5)	236 (59.1)		
Partial unilateral	11 (6.8)	60 (15.0)		
Total unilateral	13 (8.0)	72 (18.0)		
Partial bilateral	5 (3.1)	13 (3.3)		
Total bilateral	22 (13.6)	18 (4.5)		
Tumor volume, ml	11.0 ± 10.8	7.8 ± 7.0	< 0.001	1
Prostate volume, ml	37.0 ± 16.0	32.9 ± 16.2	0.007	
Length of hospital stay, d	6.5 ± 1.6	6.8 ± 1.4	0.018	

Data are given as mean ± standard deviation or as n (%). *ASA* American Society of Anesthesiologists, *CCI* Charlson Comorbity Index, *PACU* postanesthesia care unit, *UICC* Union for International Cancer Control

^a^Highest vasopressor support intraoperatively

^b^Assessed by the Numeric Rating Scale

^c^According to the TNM staging system of the American Joint Committee on Cancer

### Quality of recovery by surgical technique

There was no difference in baseline QoR-15GE scores between ORP and RARP (ORP: 139 ± 12; RARP: 139 ± 13; *p* = 0.997). QoR-15GE scores one day after surgery were lower after RARP compared to ORP (ORP: 113 ± 22; RARP: 107 ± 24; *p* = 0.006). Irrespective of whether patients underwent RARP or ORP, QoR-15GE sum scores on the first postoperative day were lower compared to baseline QoR-15GE sum scores in both groups with a higher ΔQoR-15GE in patients undergoing RARP (ORP: 26.5 ± 20; RARP: 32 ± 23; *p* = 0.006). Details on QoR-15GE scores by surgical technique between baseline assessments and postoperative day five are presented in Table 2 and Online Resource 1. Online Resource 2 shows the change from baseline QoR-15GE sum scores over time for individual patients. Mean scores for single items of the QoR-15GE by surgical technique are presented in Online Resource 3.

**Table 2:** Quality of recovery scores

	ORP (n = 162)	RARP (n = 400)	All (n = 562)	*P*	Missing (n)
QoR-15GE baseline	139 ± 12	139 ± 13	139 ± 13	0.997	5
QoR-15GE POD1	113 ± 22	107 ± 24	109 ± 23	0.006	39
QoR-15GE POD2	125 ± 19	119 ± 21	121 ± 21	0.006	58
QoR-15GE POD3	130 ± 18	127 ± 18	128 ± 18	0.140	82
QoR-15GE POD4	128 ± 23	130 ± 17	129 ± 19	0.331	201
QoR-15GE POD5	128 ± 23	131 ± 17	130 ± 18	0.486	381

Development of the sum scores from the preoperative baseline values until the 5th postoperative day. Data are given as mean ± standard deviation. *POD* Postoperative day, *QoR* Quality-of Recovery-15-score, *Sum* Sum scores

The difference in means between ORP (26.5) and RARP (32.4) was statistically significant (*p* = 0.006, 95% CI [-10.40; -1.72]). After adjustment for clinically relevant confounding variables, surgical technique remained associated with ΔQoR-15GE (Estimate 4.39; 95% CI [0.27; 8.50], *p* = 0.037); Table 3.

**Table 3:** Factors associated with quality of postoperative recovery

Predictors	Estimates	95% CI	*P*
Independent variable: decrease in quality of recovery (n = 518)			
(Intercept)	− 13.93	− 37.56; − 9.71	0.248
QoR-15GE preoperative sum score	0.33	0.19; 0.48	< 0.001
RARP (reference: ORP)	4.39	0.27; 8.50	0.037
Duration of surgery, min	0.03	− 0.01; 0.08	0.127
Nerve resection (reference: no nerve resection)	3.27	0.61; 7.14	0.098
Charlson Comorbidity Index	− 2.53	− 3.83; − 1.23	< 0.001

Multivariable linear regression model assessing the relationship between clinically relevant variables and a decline in quality of recovery (difference between the preoperative QoR-15GE sum score and the postoperative QoR-15GE sum score)

*ORP* open retropubic radical prostatectomy, *QoR-15GE* German version of the Quality-of Recovery-15 questionnaire, *RARP* robot-assisted radical prostatectomy. The analysis was performed in patients with complete assessments at baseline and on postoperative day 1 (n = 518)

### Time course of postoperative recovery by surgical technique

For postoperative day one, we observed a difference of 6.22 (95% CI [2.38; 10.06], *p* = 0.002) between ORP and RARP. The difference remained on postoperative day two (difference 5.72, 95% CI [1.84; 9.59], *p* = 0.004). For postoperative days three to five the difference between ORP and RARP became smaller (Online Resource 4). After adjustment for relevant confounding variables (‘duration of surgery’, ‘nerve resection’, ‘Charlson Comorbidity Index’), the effects on postoperative day one and two remained comparable (Online Resource 5). Linear mixed effects models with random intercepts for the interaction effect of surgical technique by postoperative day with and without adjustment for clinically relevant confounding variables are presented in Online Resources 6 and 7. Table 4 presents estimated marginal means for QoR-15GE sum scores by postoperative day.

**Table 4:** Estimated marginal mean QoR-15GE sum scores with 95% confidence intervals and p-values of the contrasts based on the linear mixed effects model

	ORP		RARP			
	Estimated marginal mean	95% CI	Estimated marginal mean	95% CI	*P*	Missing (n)
POD 1	113	110; 116	107	105; 109	0.002	39
POD 2	125	122; 128	119	117; 121	0.004	58
POD 3	130	126; 133	127	125; 129	0.132	82
POD 4	129	125; 133	131	129; 133	0.403	201
POD 5	138	132; 143	133	131; 136	0.152	381

*ORP* open retropubic radical prostatectomy, *POD* postoperative day, *QoR-15GE* German version of the Quality-of Recovery-15 questionnaire, *RARP* robot-assisted radical prostatectomy

## Discussion

The main findings of our study are: 1) Surgical technique for radical prostatectomy is significantly associated with ΔQoR-15GE. 2) Patients with RARP report lower QoR on postoperative days one and two compared to those with ORP. 3) Irrespective of surgical technique, QoR-15GE scores between postoperative days two to five are higher than those on postoperative day one. 4) The QoR-15GE plausibly reflects the quality of recovery throughout postoperative day five.

Despite similar oncological and functional urological outcomes, there is evidence supporting a superior effect of RARP over ORP on patient-reported outcomes [12]. Therefore, we had expected that patients undergoing RARP would exhibit better self-reported postoperative recovery one day after surgery compared to patients who had undergone ORP. Despite having similar baseline values preoperatively, we observed lower QoR-15GE scores after RARP compared with ORP on the first two postoperative days. Also, the multivariable analysis confirmed the strong association between surgical procedure and the quality of recovery. This is even more surprising, as patients with ORP remained longer at the postanesthesia care unit, had higher tumor volumes and tumor stage, and more extensive nerve resection compared with RARP patients.

Our findings may be attributable to factors specific for robot-assisted surgery. In addition to wound pain in the abdominal incision, patients may also encounter rib or shoulder pain following laparoscopy [19]. This discomfort arises from diaphragm irritation and typically resolves within few days. Also, it is likely that cramp-like lower abdominal pain occurs within the first hours after laparoscopic surgery [20, 21]. Postoperative pain primarily stems from the application of pneumoperitoneum [22]. Despite various measures such as warming and humidification of the insufflated gas or reduction of the intraabdominal pressure, adverse effects have been reduced but not completely prevented [22]. While visceral pain represents the primary source of pain within the first 24 h, increasing shoulder pain predominates in 35% to 63% of patients thereafter [20, 23]. Moreover, nausea and vomiting commonly occur within the first 24 h after laparoscopic procedures and are of multifactorial origin. The increase in intraabdominal pressure leads to stretching of the diaphragm, activation of mechanoreceptors, and increased serotonin release [24, 25].

Importantly, the difference in quality of recovery between surgical techniques diminishes over time and shows similar results from the third postoperative day. Thus, similar quality of recovery scores from day three underline previous findings of a lack of association between surgical technique for radical prostatectomy and patient-reported outcomes [14]. One of the largest studies to analyze the difference in quality of life outcomes between open and robot-assisted surgery was performed in Sweden [14]. In a controlled, nonrandomized design, Wallerstedt et al. assessed quality of life at 3, 12, and 24 months without reporting a difference between surgical approaches [14]. In a randomized controlled Australian trial, health-related quality of life at 6, 12, and 24 months were analyzed as secondary endpoints without showing a difference between ORP and RARP [6]. Similarly, two single center studies did not find differences in quality of life outcomes at 12 or 18 months after RARP, ORP, or laparoscopic surgery [26, 27].

While numerous studies have addressed quality of life and functional outcomes after radical prostatectomy, studies comparing overall self-reported health status in the immediate postoperative period after RARP vs ORP are limited. The postoperative QoR-15GE sum scores demonstrate an increase from the first to the third postoperative day in patients undergoing radical prostatectomy. The continuous increase in scores in both groups suggests that the QoR-15GE plausibly reflects recovery beyond the first postoperative day. This is line with findings from Lyckner et al., who observed an increasing trend of the Swedish version of the QoR-15 beyond day one after elective non-cardiac surgery [28]. Overall, the multivariable regression model provides comprehensive explanation for the observed variability in postoperative QoR-15GE sum scores, highlighting the complex interplay of surgical factors, patient characteristics, and temporal effects on recovery outcomes. These findings suggest that the QoR-15GE, although previously validated for use on the first postoperative day, may be appropriate for serial assessments and may also accurately reflect recovery beyond postoperative day one.

### Limitations and strengths

Several limitations of this cohort study need to be addressed. First, while this research was conducted at the Martini Clinic, which is one of the largest prostate cancer centers worldwide, the external validity of our findings is constrained by the fact that we analyzed data from one single center. Consequently, the generalizability of our results to other patient cohorts or research sites may be limited. Second, we present findings from an observational study that compared self-reported outcomes during the early postoperative period between two surgical techniques. Although we tried to control for relevant confounding variables including comorbid conditions, the duration of surgery, and nerve resection, we may have missed unmeasured variables confounding our results. Third, the proportion of completed questionnaires declined gradually from postoperative day one to five. The substantial decrease in questionnaire completion from the fourth postoperative day onwards might be attributable to the fact that patients are typically discharged on the fourth postoperative day and did not fill out the questionnaire on the day of discharge. Furthermore, patients may experience increasing loss of motivation during their inpatient stay.

Nevertheless, we observed that the acceptability and feasibility of using the QoR-15GE for repeated assessments remained relatively high throughout the study period.

## Conclusions

In this prospective cohort study, we found a significant association between the surgical approach for radical prostatectomy and self-reported quality of recovery. While patients undergoing RARP report lower postoperative recovery until postoperative day two compared with those undergoing ORP, measures of postoperative recovery realign starting from day three. The acceptability and feasibility of using the QoR-15GE remained high throughout serial measurements until hospital discharge. The QoR-15GE demonstrates robustness, expanding its applicability beyond the immediate postoperative period.

## Supplementary Information

Below is the link to the electronic supplementary material.

Supplementary file1 (DOCX 116 KB)

## Data Availability

The datasets used and/or analyzed during the current study are available from the corresponding author on reasonable request.

## Abbreviations

ORP: Open retropubic radical prostatectomy
QoR-15: Quality of Recovery 15 questionnaire
QoR-15GE: German version of the QoR-15
ΔQoR-15GE: Difference between the QoR-15GE at baseline (preoperative assessment) and at day one after surgery
RARP: Robot-assisted radical prostatectomy
