# Supplementary material for: Self-reported quality of recovery after radical prostatectomy—a prospective cohort study
Source: Qual Life Res. 2025 Jul 18;34(10):2911–9. doi: 10.1007/s11136-025-04026-6 (PMC12535498; doi:10.1007/s11136-025-04026-6)
Supplement: Supplementary file 1 — Supplementary file1 (DOCX 116 KB) [file 11136_2025_4026_MOESM1_ESM.docx]

# **Title**

Self-reported quality of recovery after radical prostatectomy – a prospective cohort study

**Journal name**

Quality of Life Research

**Authors**

Marlene Fischer MD, PhD^1,2§^*, Josephine Küllmei^1§^, Linda Krause PhD^3^, Peipei Wei MSc^1^, Ursula Kahl MD^1^, Elena Kainz MD^1^, Caspar Mewes MD^1^, Markus Graefen MD^4^, Alexander Haese MD^4^, Christian Zöllner MD^1^, Lili Plümer MD^1^

§The authors contributed equally to this work.

**Affiliations**

^1^Department of Anesthesiology, University Medical Center Hamburg-Eppendorf, Hamburg, Germany; ^2^Department of Intensive Care Medicine, University Medical Center Hamburg-Eppendorf, Hamburg, Germany; ^3^Institute of Medical Biometry and Epidemiology, University Medical Center Hamburg-Eppendorf, Hamburg, Germany; ^4^Martini-Klinik, Prostate Cancer Center, University Medical Center Hamburg-Eppendorf, Hamburg, Germany.

***Corresponding author**

Marlene Fischer, MD, PhD, Department of Intensive Care Medicine, University Medical Center Hamburg-Eppendorf, Martinistrasse 52, 20246 Hamburg, Germany; Phone: +49 15222827500; Email: [mar.fischer@uke.de](mailto:mar.fischer@uke.de). ORCID: 0000-0001-7530-8155

**Supplementary Material**

**Online Resource 1**

**Online Resource 1:** Mean QoR-15GE scores in patients with open retropubic (ORP) and robot-assisted (RARP) radical prostatectomy between preoperative assessments (baseline) and postoperative day (POD) five. QoR-15GE: German version of the quality-of-recovery-15 score.

**Online Resource 2**

*
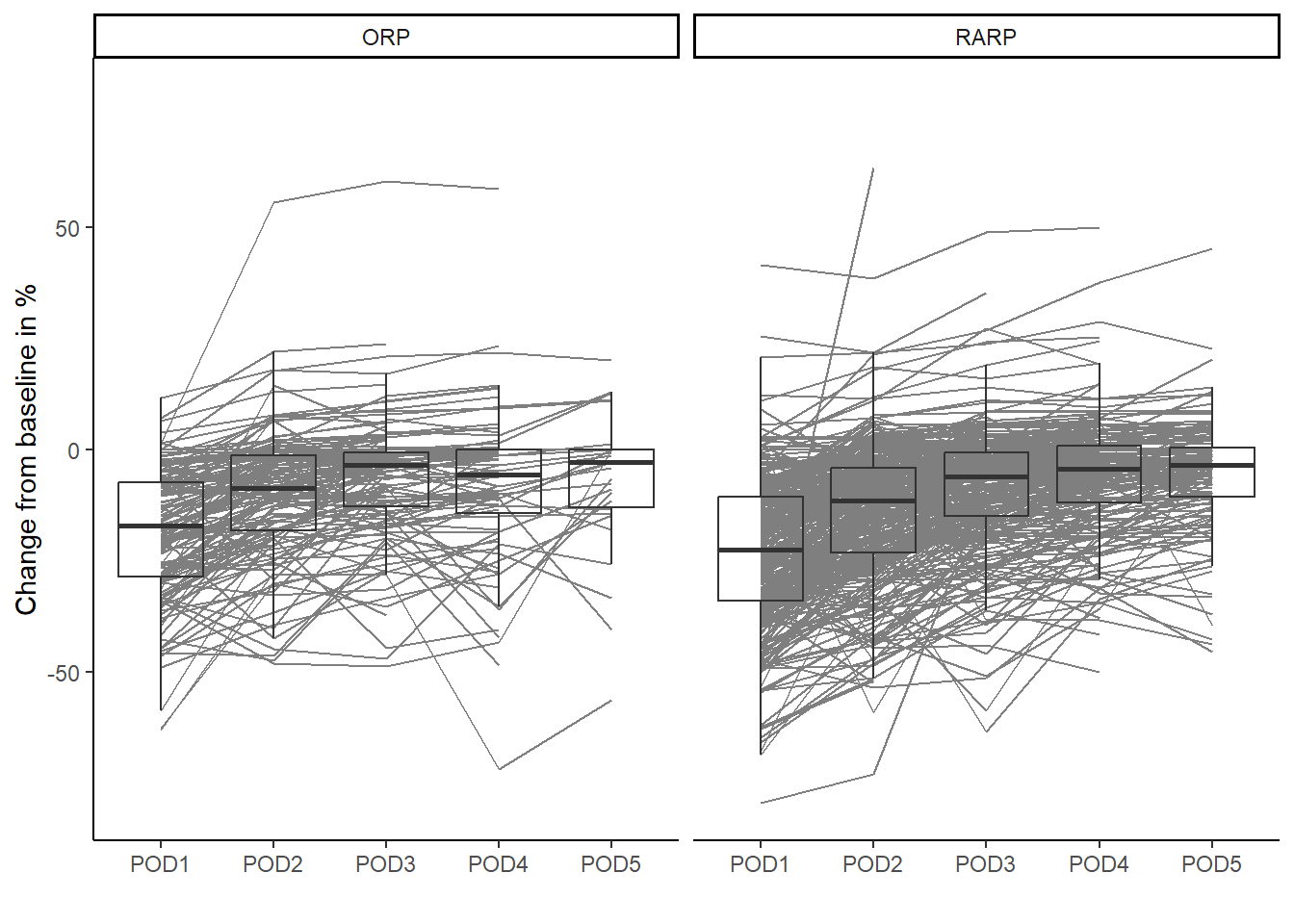
*

**Online Resource 2**: Difference between preoperative and postoperativ8e assessments of quality of recovery with the German version of the Quality of recovery-15 questionnaire (QoR-15GE). The change from baseline QoR-15GE sum scores is presented for individual patients between postoperative day (POD) 1 to 5, stratified by surgical technique. ORP: open retropubic radical prostatectomy. RARP: robot-assisted radical prostatectomy. Grey lines denote individual change from baseline scores in %.

**Online Resource 3**


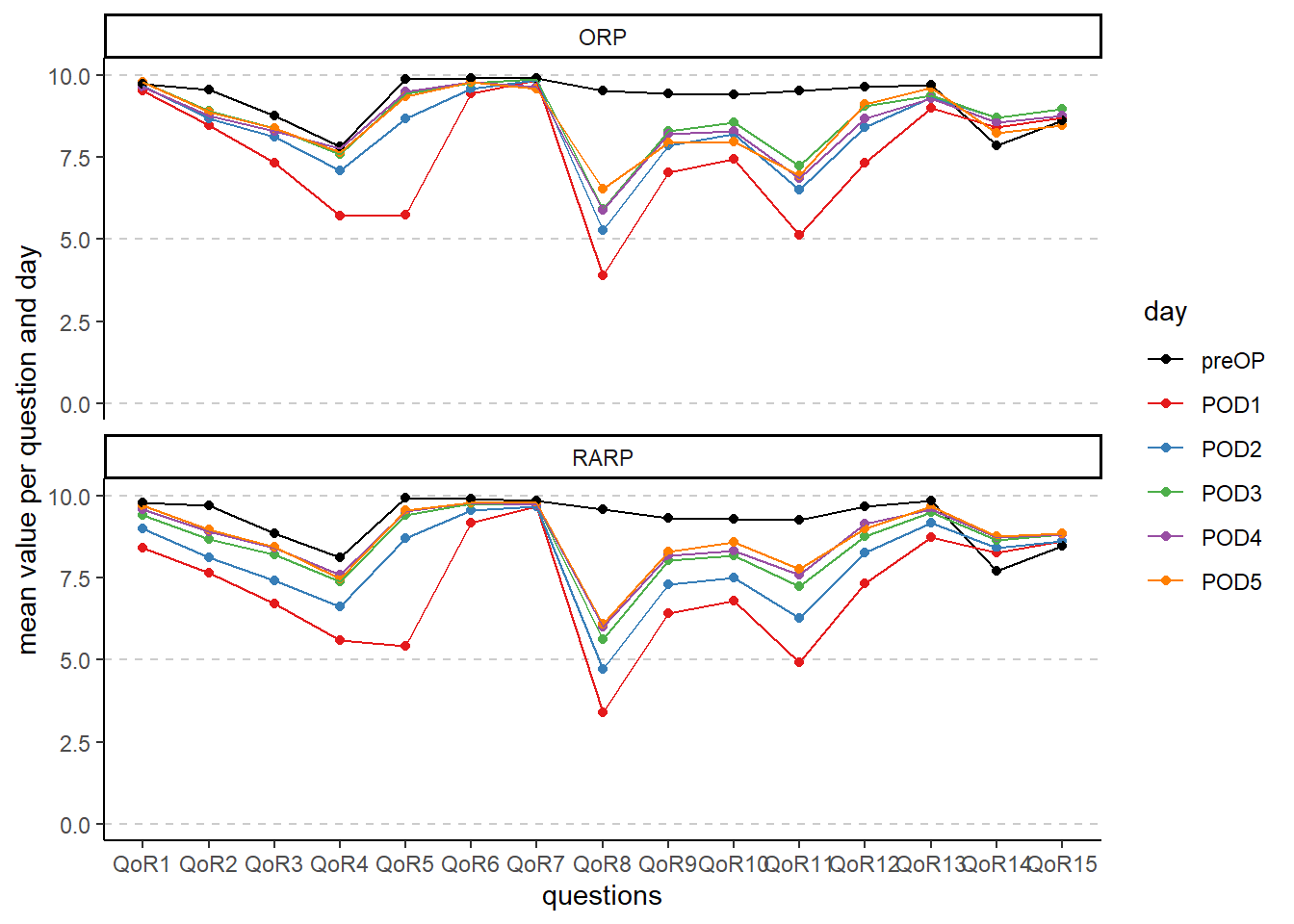


**Online Resource 3**: Mean scores of single items of the German version of the Quality of recovery-15 questionnaire by surgical technique. ORP: open retropubic radical prostatectomy. POD: postoperative day. RARP: robot-assisted radical prostatectomy.

**Online Resource 4**

| **Contrast RARP - ORP** | **Estimate** | **95% CI** | ***P*** |
| --- | --- | --- | --- |
| POD 1 | -6.22 | -10.06; -2.38 | 0.002 |
| POD 2 | -5.72 | -9.59; -1.84 | 0.004 |
| POD 3 | -3.01 | -6.93; 0.91 | 0.132 |
| POD 4 | 1.82 | -2.45; 6.09 | 0.403 |
| POD 5 | -4.23 | -10.02; 1.56 | 0.152 |

**Online Resource 4**: Marginal means with 95% confidence intervals (CI) for the difference in QoR-15GE sum scores between open (ORP) and robot-assisted radical prostatectomy (RARP) on postoperative days (POD) one to five. QoR-15GE: German version of the quality-of-recovery-15 score.

**Online Resource 5**

| **Contrast RARP - ORP** | **Estimate** | **95% CI** | ***P*** |
| --- | --- | --- | --- |
| POD 1 | -5.54 | -9.42; -1.66 | 0.005 |
| POD 2 | -5.03 | -8.94; -1.12 | 0.012 |
| POD 3 | -2.31 | -6.27; 1.65 | 0.252 |
| POD 4 | 2.51 | -1.80; 6.82 | 0.254 |
| POD 5 | -3.46 | -9.29; 2.36 | 0.244 |

**Online Resource 5**: Marginal means with 95% confidence intervals (CI) for the difference in QoR-15GE sum scores between open (ORP) and robot-assisted radical prostatectomy (RARP) on postoperative days (POD) one to five. QoR-15GE: German version of the quality-of-recovery-15 score. Results are adjusted for clinically relevant confounding variables (‘duration of surgery’, ‘nerve resection’, ‘Charlson Comorbidity Index’).

**Online Resource 6**

| **Predictors** | **Estimates** | **95% CI** | ***P*** |
| --- | --- | --- | --- |
| (Intercept) | 113.17 | 109.93 – 116.40 | <0.001 |
| QoR-15GE sum score (POD 2) | 11.68 | 9.11 – 14.26 | <0.001 |
| QoR-15GE sum score (POD 3) | 16.50 | 13.87 – 19.13 | <0.001 |
| QoR-15GE sum score (POD 4) | 15.91 | 12.85 – 18.96 | <0.001 |
| QoR-15GE sum score (POD 5) | 24.40 | 19.62 – 29.18 | <0.001 |
| RARP (reference: ORP) | -6.22 | -10.06 – -2.39 | 0.001 |
| ORP * QoR-15GE sum score (POD 2) | 0.51 | -2.55 – 3.57 | 0.745 |
| ORP * QoR-15GE sum score (POD 3) | 3.21 | 0.09 – 6.33 | 0.044 |
| ORP * QoR-15GE sum score (POD 4) | 8.05 | 4.49 – 11.60 | <0.001 |
| ORP * QoR-15GE sum score (POD 5) | 1.99 | -3.30 – 7.28 | 0.460 |

**Online Resource 6**: Linear mixed effects model with random intercept for the interaction effect of surgical technique by postoperative day (POD). QoR-15GE: German version of the quality-of-recovery-15 score.

**Online Resource 7**

| **Predictors** | **Estimates** | **95% CI** | ***P*** |
| --- | --- | --- | --- |
| (Intercept) | 114.93 | 106.01; 123.86 | <0.001 |
| Duration of surgery, min | -0.05 | -0.08; -0.01 | 0.014 |
| Nerve resection (reference: no nerve resection) | -0.28 | -3.52; 2.95 | 0.863 |
| Charlson Comorbidity Index | 1.20 | 0.11; 2.28 | 0.030 |
| QoR-15GE sum score (POD 2) | 11.69 | 9.11; 14.27 | <0.001 |
| QoR-15GE sum score (POD 3) | 16.48 | 13.85; 19.11 | <0.001 |
| QoR-15GE sum score (POD 4) | 15.90 | 12.84; 18.95 | <0.001 |
| QoR-15GE sum score (POD 5) | 24.31 | 19.53; 29.09 | <0.001 |
| RARP (reference: ORP) | -5.54 | -9.42; -1.67 | 0.005 |
| ORP * QoR-15GE sum score (POD 2) | 0.51 | -2.55; 3.58 | 0.742 |
| ORP * QoR-15GE sum score (POD 3) | 3.23 | 0.11; 6.36 | 0.042 |
| ORP * QoR-15GE sum score (POD 4) | 8.05 | 4.50; 11.61 | <0.001 |
| ORP * QoR-15GE sum score (POD 5) | 2.08 | -3.21; 7.37 | 0.441 |

**Online Resource 7**: Linear mixed effects model with random intercept for the association between the interaction of surgical technique by postoperative day (POD), adjusted for clinically relevant confounding variables. QoR-15GE: German version of the quality-of-recovery-15 score.
